# 3D Perfusable Hydrogel Recapitulating the Cancer Dynamic Environment to in Vitro Investigate Metastatic Colonization

**DOI:** 10.3390/polym12112467

**Published:** 2020-10-24

**Authors:** Chiara Vitale, Arianna Fedi, Alessandra Marrella, Gabriele Varani, Marco Fato, Silvia Scaglione

**Affiliations:** 1 National Research Council of Italy, Institute of Electronic, Computer and Telecommunications (IEIIT) Institute, 16149 Genoa, Italy; chiara.vitale@ieiit.cnr.it (C.V.); arianna.fedi@ieiit.cnr.it (A.F.); vgabri89@gmail.com (G.V.); marco.fato@unige.it (M.F.); silvia.scaglione@ieiit.cnr.it (S.S.); 2 Department of Computer Science, Bioengineering, Robotics and Systems Engineering, University of Genoa, 16126 Genoa, Italy

**Keywords:** hydrogels, fibrin, alginate, CFD, metastasis, extravasation

## Abstract

Metastasis is a dynamic process involving the dissemination of circulating tumor cells (CTCs) through blood flow to distant tissues within the body. Nevertheless, the development of an in vitro platform that dissects the crucial steps of metastatic cascade still remains a challenge. We here developed an in vitro model of extravasation composed of (i) a single channel-based 3D cell laden hydrogel representative of the metastatic site, (ii) a circulation system recapitulating the bloodstream where CTCs can flow. Two polymers (i.e., fibrin and alginate) were tested and compared in terms of mechanical and biochemical proprieties. Computational fluid-dynamic (CFD) simulations were also performed to predict the fluid dynamics within the polymeric matrix and, consequently, the optimal culture conditions. Next, once the platform was validated through perfusion tests by fluidically connecting the hydrogels with the external circuit, highly metastatic breast cancer cells (MDA-MB-231) were injected and exposed to physiological wall shear stress (WSS) conditions (5 Dyn/cm^2^) to assess their migration toward the hydrogel. Results indicated that CTCs arrested and colonized the polymeric matrix, showing that this platform can be an effective fluidic system to model the first steps occurring during the metastatic cascade as well as a potential tool to in vitro elucidate the contribution of hemodynamics on cancer dissemination to a secondary site.

## 1. Introduction

Circulating tumor cells (CTCs) are viable cells that circulate in the bloodstream after detaching from a primary tumor [1]. Over the past decade, different studies showed that these cells play a key role in tumor progression [2]. In fact, it was shown a correlation between the presence of circulating tumor cells with a short survival rate of patients with metastatic carcinoma [3,4]. 

During the process of metastasis, tumor cells intravasate into bloodstream and lymphatic vessels, translocate until reaching distant tissues, to then extravasate, attach and proliferate, giving origin to a metastatic site [5]. 

In this context, an improved understanding of the mechanical forces encountered by CTCs in the blood flow is crucial for fully decoding the various process of metastatic cascade and delineating vulnerable CTCs states for therapeutic intervention [6]. In fact, CTCs must be able to withstand different fluid-dynamical stresses during their circulation, in order to survive and to adhere to the endothelial wall of the potential metastatic site, to finally extravasate to origin a secondary tumor [7]. 

Currently, conventional studies of extravasation rely primarily on in vivo models [8]. Although these are considered the most physiologically reliable models for extravasation, they present some limitations, such as poor segmentation of the essential steps of metastatization and hard capture of the individual variables which affect the whole process. Therefore, to overcome these disadvantages, some micro-fluidic systems (called lab-on-chip) have been employed to investigate the metastatic cascade, by manipulating small volumes of cellular material and reproducing the dynamic interactions between tumor cells and the ECM microenvironment (e.g., CTCs arrest and transmigration into a secondary site) [9,10,11]. 

For example, Jeon et al. fabricated a microfluidic device to investigate the cancer extravasation by analyzing the tumor cell adhesion and then the transmigration across an endothelial monolayer. In particular, they used a high-resolution imaging technique in order to detect and quantify the number of cells involved in these processes [12]. In addition, in another work, Jeon et al. realized a microfluidic device to study human metastatic breast cancer cell extravasation within a perfusable bone-mimicking microenvironment [13]. The resulting device represented a functional human multi-culture device where breast cancer cells could flow, adhere, and metastasize through a micro-vascular network. Moreover, in a further study Ma et al. investigated the breast cancer (MDA-MB-231 and MCF-7) cell extravasation toward different cell-based spheroids, by realizing a PDMS-based microfluidic device containing a Boyden-chamber-like system [14]. 

However, these lab-on-chip devices show some limitations, due to their small dimensions, poor cells number and limited liquid volumes used. In fact, the over-miniaturization of these models could impair the realism of the tumor microenvironment resembled in vitro [15]. 

In particular, the attachment and colonization of CTCs to a secondary tissue require a proper interaction with a surrounding extracellular matrix (ECM) [16]. In fact, once CTCs mechanically arrest close to the target tissues, the subsequent establishment of the metastasis and cells growth is highly dependent on the interaction between metastatic cancer cells and the surrounding environment [17]. In this context, the progress in tissue engineering led to design matrix-based culture systems that recapitulate the biological, biophysical and biochemical environment of the natural extracellular matrix [18,19]. Specifically, hydrogels have been widely used to model the 3D ECM, because of their advantageous properties similar to those of the native one [20,21]. In fact, they possess a high-water content, which provides biochemical and biomechanical cues similar to the physiological ECM [21,22,23,24]. 

Presently, vascularized 3D hydrogels are increasingly gained attention to investigate the dynamic cancer-associated phenomena. In particular, vasculogenesis-based, subtractive and bioprinter-based models can be used to realize channels within polymeric matrices [12,25,26]. Among them, vasculogenesis models allow obtaining a vascularization through a growth factors gradient, thus inducing endothelial cells (ECs) to form a capillary-like network. These are the commonest methods used to create vascularized 3D hydrogel-based models to study cancer cells extravasation, as it occurs at the capillary level. For example, Chen et al. realized a two-channels device to co-culture human umbilical vein endothelial cells (HUVECs) and lung fibroblasts stimulated with inflammatory cytokines within a 3D fibrin ECM. After 2 days of culture, HUVECs organized into tubular structures characterized by tight junctions and a physiological vessels permeability [27]. However, injecting fluid flow resembling the bloodstream and controlling its biophysical properties, crucial for cancer cells migration and invasion, result in being very difficult because syringe or peristaltic pumps cannot be directly connected to the inner vasculature [28]. On the other hand, although bioprinting techniques allow creating more complex and variable sized networks, they are often hardly accessible due to the high costs. 

Taking together all these considerations, in this work a fluid-dynamic perfusable hydrogel-based system was fabricated, through a simple and low-cost, but effective, subtractive method, as model of cancer cells extravasation. In particular, we developed a hydrogel-based 3D fluidic system, representative of the bloodstream, where CTCs can experience physiologically relevant forces within an “ECM-like” environment, as it happens in vivo. Specifically, a hydrogel matrix with a vessel-mimicking channel was realized and directly connected with an external fluidic system, where it is possible to set and precisely monitor fluid flow patterns. Two kinds of hydrogels were tested and compared: alginate and fibrin. Alginate is widely employed in many different biomedical applications, such as tissue engineering, tissue modeling and drug delivery [29,30,31]. Moreover, it was chosen because it is bio-inert, easy-to-use, cheap and has tunable mechanical properties [32,33]. Likewise, fibrin is extensively adopted in several tissue engineering fields, such as bone regeneration [34] as well as for cardiovascular applications [35,36,37], because of its higher biocompatibility and bioactivity [38]. 

CFD simulations were performed to quantify the fluid-dynamic stimuli at tissue scale, to predict the hydrogel mechanical response to fluid flow-associated forces, as well as to evaluate if an adequate nutrients fluxes were properly provided to the 3D hydrogels [39]. In particular, the glucose transport was modeled, as it is a fundamental nutrient for cancer cells metabolism and activity [40]. 

Finally, the 3D fluid-dynamic hydrogel-based system was validated with perfusion tests to experimentally demonstrate the performance of the system.

In addition, highly metastatic breast cancer cells (MDA-MB-231) were injected in the circuit, flowing under WSS conditions, and their viability upon circulation as well as their capability to invade the surrounding ECM-like hydrogel were assessed, finally mimicking one of the crucial steps of the metastatic cascade.

## 2. Materials and Methods 

### 2.1. Channel-Based Hydrogels Realization

Firstly, we realized a mold through 3D printing technology (Form 2, Formlabs, Lecco, IT) composed by a sphere (diameter = 1 cm), where the hydrogel representing the metastatic tumor mass was placed, and two lateral cylinders to easily connect the hydrogel spherical central body to an external fluidic circuit. The mold was fabricated with a biocompatible resin (Dental LT Resin, Formlabs, Lecco, IT) by using a resolution of 100 µm. After printing, it was washed with isopropyl alcohol for 5 min and then photocured through UV light for 20 min to eliminate resin residues and to optimize the photo-linking process, respectively. 

Two distinct materials were selected to prepare the hydrogels: alginate and fibrin. For each polymer and cross-linking agent, different concentrations and precursor solution/cross-linking agent ratios were tested. Briefly, alginate powder (Manugel GMB, FMC Biopolymer, Girvan, UK) was dissolved in physiological solution (0.9% *w*/*v* NaCl) at the final concentration of 1% *v*/*v*, 1.5% *v*/*v*, 2% *v*/*v* and 2.5 *w*/*v*; then, the hydrogel were crosslinked into the mold by adding a calcium chloride solution (CaCl_2_), by varying three concentrations: 0.5 M, 0.75 M and 1 M. Moreover, CaCl_2_/alginate 1:1 *v*/*v*, 1:3 *v*/*v* and 1:6 *v*/*v* ratios were assessed. The cross-linking occurred after 15 min at room temperature (25 °C). 

Fibrin hydrogel was realized by combining fibrinogen from human plasma (F3879 Sigma Aldrich, Saint Louis, MO, USA) (20 mg/mL or 40 mg/mL) diluted in phosphate buffered saline solution (PBS)) with thrombin from human plasma (T6884 Sigma Aldrich, Saint Louis, MO, USA) (25 U/mL or 10 U/mL) diluted in CaCl_2_ 5 mM solution. Furthermore, we tested the thrombin/fibrinogen ratio from 1:1 *v*/*v* to 1:3 *v*/*v*. The cross-linking occurred after 15 min at 37 °C. 

The channel was formed by inserting a 21-gauge needle (800 μm) inside the mold, where the polymeric solutions were added, respectively. The cross-linking agents were poured within the mold and, after cross-linking, the needle was gently removed, leaving a hollow round canal within the gel. 

### 2.2. Channel-Based Hydrogels Characterization

#### 2.2.1. Diffusion Measurements

Glucose diffusion coefficients in alginate and fibrin hydrogels were experimentally measured. Hydrogels were formed by pouring alginate and fibrin in a CaCl_2_ and thrombin bath, respectively. Then, hydrogel beads were soaked in 4 mL of deionized water containing glucose at the initial concentration of 200 mg/mL and 1 mL of solution was sampled every hour for 8 h. Glucose absorbance was red at 193 nm by using a spectrophotometer and the glucose diffusivity within the two hydrogels was calculated through the best fitting of the data obtained from the experimental release studies. Briefly, a simulation of the mass transport process occurring between the medium and the hydrogels was performed by employing the Transport of the diluted species (TDS) module of Comsol Multiphysics (COMSOL AB, Stockholm, Sweden) in order to fit the raw data (not shown) and thus to get the optimal glucose diffusion coefficient by comparing the two resulting curves. The implemented model in Comsol Multiphysics was based on the second Fick law assuming that only the diffusion process in the bath could occur:

(1) $$\frac{\delta c}{\delta t}+\nabla \cdot \left(J\right)=0\;$$

where c is the component concentration and J is the mass diffusive flux vector, defined by the Fick law as:

(2) J=−D ∇c 

where D is the diffusion coefficient of the metabolite.

Experiments were performed in triplicates and the results were expressed as mean and standard deviation.

#### 2.2.2. Mechanical Characterization

Both hydrogels were mechanically tested through a dynamic mechanical analysis (DMA) at the physiological frequency of 1 Hz. Briefly, the instrument was composed by a plate supporting the sample, a mini-shaker that generate a vertical oscillation, connected to a cylindrical indenter (diameter = 5 mm), which thus apply a sinusoidal stress on the cylindrical sample (diameter = 5 mm, height = 3 mm), linked with a force transducer, and finally by a laser measuring the displacement. Before all tests, samples were 10% pre-deformed. Measurements were recorded in triplicate and the results were expressed as mean and standard deviation. When the stress is applied the following equations hold:

(3) $$\{\begin{array}{c} \sigma =\sigma_{0}sin\omega t\\ \varepsilon =\varepsilon_{0}sin\omega t+\delta \end{array}$$

where *σ* is the stress tensor, *ε* the strain tensor, ω the frequency, *t* the time and *δ* the lag. 

The storage modulus E (or Young modulus) measuring the stored energy and representing the elasticity of the material, and the loss modulus $E^{\prime \prime }$ (or viscous modulus) measuring the dissipated energy as heat and representing the viscosity, have been evaluated and defined as follows:

(4) $$\;\;\{\begin{array}{c} E=\frac{\sigma_{0}}{\varepsilon_{0}}cos\delta \\ E^{\prime \prime }=\frac{\sigma_{0}}{\varepsilon_{0}}sin\delta \end{array}$$

The damping properties were also analyzed by calculating the loss factor (tanδ), as ratio between the viscous modulus over the storage one [41]:

(5) $$\tan\delta =\frac{E^{\prime \prime }}{E}$$

Furthermore, considering the rubber elasticity theory, the following equation defines the gel crosslink density *ρ_x_* as [20]:

(6) $$\rho_{x}=\;\frac{E}{3RT}$$

where *R* is the universal gas constant (8.314 J K^−1^mol^−1^), *T* the absolute temperature (298.15 K).

#### 2.2.3. CFD analysis of the Fluid-Hydrogels Interactions 

Firstly, in order to obtain desired WSS range values inside the channel (1–10 Dyn/cm^2^) a theoretical analysis, assuming (i) a laminar flow regime, (ii) an incompressible Newtonian fluid, was carried out. Based on these hypotheses, the inverse formula of Poiseuille’s law for a cylindric tube is:

(7) $$Q=\;\frac{\pi r^{3}\tau }{4\mu }$$

where Q is the flow rate, r the channel radius, τ the shear stress and *μ* is the fluid dynamic viscosity of water.

Next, the fluid-dynamics inside the channel were simulated. This analysis was performed using the Single-Phase Laminar Fluid Flow model of Comsol Multiphysics 5.5 assuming (i) a laminar flow regime, (ii) an incompressible Newtonian fluid. The solving equations are the Navier-Stokes ones for conservation of momentum (8a) and the continuity one for conservation of mass (8b):

(8) $$\{\begin{array}{c} \rho \left[\frac{\partial u_{f}}{\partial t}+u_{f}\left(\nabla u_{f}\right)\right]=-\nabla p+\mu \left(\nabla^{2}u_{f}\right)\;\;\;\;\;\;\left(a\right)\\ \left(\nabla u_{f}\right)=0\;\;\;\;\;\;\;\;\;\;\;\;\;\;\;\;\;\;\;\;\;\;\;\;\;\;\;\;\;\;\;\;\;\;\;\;\;\;\;\;\;\;\;\;\;\;\;\;\;\;\;\;\;\;\;\;\;\;\left(b\right) \end{array}$$

where $u_{f}$ is the fluid velocity and p the pressure across the circuit. The density *ρ* (1000 kg/m^3^) and viscosity *μ* (10^−3^ Pa·s) values of water at room temperature (25 °C) were used. A flow rate was set as input according to Equation (7) to create a pressure gradient within the channel, in order to generate the fluid motion, whereas as output the atmospheric pressure was set as null, avoiding a backflow. A no-slip boundary condition was set, thus assuming no flow across channel walls. 

Moreover, the shear stress distribution within the channel was estimated. Since the steady state flow is reached almost instantaneously for our flow rate range, we considered only a steady state analysis. Indeed, an iterative geometric multigrid (GMRES) algorithm was used to solve the equations. The interaction between the fluid and the hydrogel solid structure, including both fluid pressure and viscous forces, was investigated. According to the Fluid-Structure Interaction (FSI) Multiphysics module, the interplays between the fluid (cell culture medium) and a deformable solid (3D hydrogel) were examined. In particular, the fluid-dynamic load on the structure, i.e., the hydrogel, especially focusing on the channel deformation in order to determine the fluid flow-associated forces effects on the polymer matrix, was analyzed. According to the DMA results (Table 1), the solid structure was approximated to an isotropic linear elastic material, since the elastic component was found to be predominant respect to the viscous one. Therefore, the multiphysics model equations are:

- Equation of motion, an expression of Newton’s second law:

(9) $$\nabla \cdot \sigma_{s}+F_{v}=\rho_{h}\frac{\partial u_{2}}{\partial t^{2}}$$

where $\sigma_{s}$ is the Cauchy stress tensor, *F_v_* the body force per unit volume, *ρ_h_* is the hydrogel density and *u_2_* the channel displacement vector;

- Strain-displacement equation:

(10) $$\varepsilon =\frac{1}{2}\left[\nabla u_{2}+{\left(\nabla u_{2}\right)}^{T}\right]$$

where ε is the strain tensor;

- Constitutive law for the structural material:

(11) $$\sigma_{s}=\left[\frac{E\upsilon }{\left(1+\upsilon \right)\left(1-2\upsilon \right)}\nabla \cdot u_{2}\right]I+\left[\frac{E}{1+\upsilon }\right]\varepsilon$$

where *E* is the Young Modulus, measured through DMA analysis, and υ the Poisson coefficient;

- Equation of normal components, which allows coupling the fluid-dynamics with the solids mechanics:

(12) $$\sigma_{s}\cdot n=\sigma_{f}\cdot n$$

where *n* is the normal vector on the channel walls and $\sigma_{f}$ the fluid stress tensor;

- Constitutive law for a Newtonian fluid:

(13) $$\sigma_{f}=-p_{f}I+\mu \left[\nabla u_{f}+{\left(\nabla u_{f}\right)}^{T}\right]-\frac{2}{3}\mu \left(\nabla \cdot u_{f}\right)I$$

As initial condition, the channel displacement was assumed null.

#### 2.2.4. Cell Viability Analysis within Channel-Based Hydrogels

Alginate and fibrin hydrogels were tested and compared to model the ECM environment mimicking the metastatic site. Breast cancer cells from adenocarcinoma (MDA-MB-231) were embedded within the two polymers and cultured in Dulbecco’s Modified Eagle’s Medium (DMEM; Invitrogen, Carlsbad, CA, USA) enriched with 10% Fetal Bovine Serum (FBS), 1% L-glutamine and 1% penicillin/streptomycin (all from Sigma Aldrich, Saint Louis, MO, USA) at the concentration of 2·10^6^ cells/mL. After 24 h, the cells viability was evaluated by washing the hydrogels three times with PBS and by incubating them in 2 mM calcein AM and in 4 mM EthD-1 in PBS for 15 min at 37 °C in a dark environment, to identify live and dead cells, respectively. All images were obtained using fluorescence microscope (Nikon H550L, Nikon, Tokyo, Japan) and processed with ImageJ^®^ software. Cell viability is derived as ratio between alive cells and the total number of cells for image. Measurements were recorded in triplicates and the results were expressed as mean and standard deviation. 

### 2.3. Channel-Based Hydrogels Validation under Dynamic Conditions

#### 2.3.1. Perfusion Tests

Perfusion tests were carried out to verify the two materials capability to resist to the fluid passage, previously examined through FSI analysis, and thus the fluidic continuity between the hydrogel inner channel and an external fluidic circuit. This circuit was composed by silicone tubes connected to a syringe pump (Harvard apparatus PHD2000, Holliston, MA, USA), which allowed to accurately control the flow rate and keep it constant. The pump flow rate was set in the range predicted by the CFD analysis to obtain a physiological WSS values in the 1–10 Dyn/cm^2^ range. Furthermore, we injected a dye into the entire fluid circuit and dipped the hydrogel into a colored PBS bath, to identify any liquid leakage from the channel. 

#### 2.3.2. Computational Glucose Transport Simulation

A glucose mass transport analysis was carried out, by using the TDS Multiphysics module. In addition to the diffusive mechanism, other two processes were considered: the convection transport, due to the presence of a velocity field (*u_f_*), and the metabolite consumption/production because of the cellular respiration. The glucose metabolism is accurately described by the Michaelis-Menten reaction:

(14) $$R=\frac{V_{max}c}{K_{m}+c}$$

where R is the reaction term, c is the concentration of the component, $V_{max}$ represent the maximum consumption/production rate (equal to 2.57 × 10^−4^ mol/m^3^·s) and $K_{m}$ represents the component concentration when the rate is *V_max_*/2 (equal to 2.9 mol/m^3^) [42]. 

Thus, the general form to describe the mass transport can be written as:

(15) $$\frac{\delta c}{\delta t}+\nabla \cdot \left(J\right)+u_{f}\cdot \nabla c=R\;\;\;\;\;\;\;\;\;\;\;\;\;\;$$

where J is the mass diffusive flux vector previously defined Equation (2). 

In particular, this study was conducted by considering two opposite scenarios to monitor the presence of concentration gradients inside the hydrogels: (i) a continuous refilling of the culture medium within the culture chamber and (ii) no culture medium refilling, both in static and dynamic conditions. In both cases, 5.5 mol/m^3^ was set as culture medium initial glucose concentration.

#### 2.3.3. Metastatic Breast Cancer Cells Circulation 

The mold was sterilized in ethanol 70% *v*/*v* for 30 min, washed with sterile DI water and connected to the syringe pump using autoclaved connectors (Bio-rad, Milan, IT) and Tygon tubes (i.d. = 800 μm; Saint-Gobain, Courbevoie, France). Fibrin hydrogels were prepared by using the protocol described above. The tubes inner surface was treated with 1% Pluronic F-127 (Sigma Aldrich, Saint Louis, MO, USA) for 30 min to reduce cell adhesion during circulation.

Highly metastatic breast cancer cells (MDA-MB-231) from breast adenocarcinoma were expanded in DMEM enriched with 10% FBS, 1% L-glutamine and 1% penicillin/streptomycin (all from Sigma Aldrich, Saint Louis, MO, USA). Cells were enzymatically detached with 0.05% trypsin, counted, and injected within the circuit at a density of 1·10^5^ cells/mL. The pump flow rate was set to 1.5 mL/min to reproduce the physiological WSS value of 5 Dyn/cm^2^. As controls, MDA-MB-231 cells were injected in a circuit composed of Tygon tubes (i.d. = 800 μm) where cells could circulate at the same WSS and cultured in static conditions over surfaces pre-treated with Pluronic.

#### 2.3.4. Recovered Cells Viability Assay and Cells Migration in the Hydrogel under Circulation

Cells viability was analyzed after 24 h of circulation by using the Live/Dead assay (Sigma Aldrich, Saint Louis, MO, USA). Shortly, MDA-MB-231 were recovered from the circuits (petri dish for the static control) and counted. 

Then, cells were placed in 96 well plates, where they could adhere. After 24 h, supernatant was collected to count the cells which did not adhere on the plastic surfaces, while the remaining cells were incubated with 2 mM calcein AM and in 4 mM EthD-1 in PBS for 15 min at 37 °C in a dark environment, to identify live and dead cells, respectively. Cells were washed three times in PBS and observed under microscope. All images were obtained by using fluorescence microscope (Nikon H550L, Nikon, Tokyo, Japan) and processed with ImageJ^®^ software. Cells’ viability was derived as the ratio between the number of alive cells and the total number of cells for each picture.

A toluidine blue staining on the hydrogel was performed to establish the presence of the cells within the hydrogel after 24 h of circulation. Briefly, fibrin hydrogel was fixed in 4% paraformaldehyde and next washed three times with PBS. Then, the gel was immersed in a 0.2% toluidine blue (Sigma Aldrich, Saint Louis, MO, USA) solution for 15 min and, subsequently, washed in PBS until the excess toluidine was removed. Images were captured using light transmitted microscope (Nikon H550L, Nikon, Tokyo, Japan). 

## 3. Results

### 3.1. Hydrogels Realization

**Figure 1:**
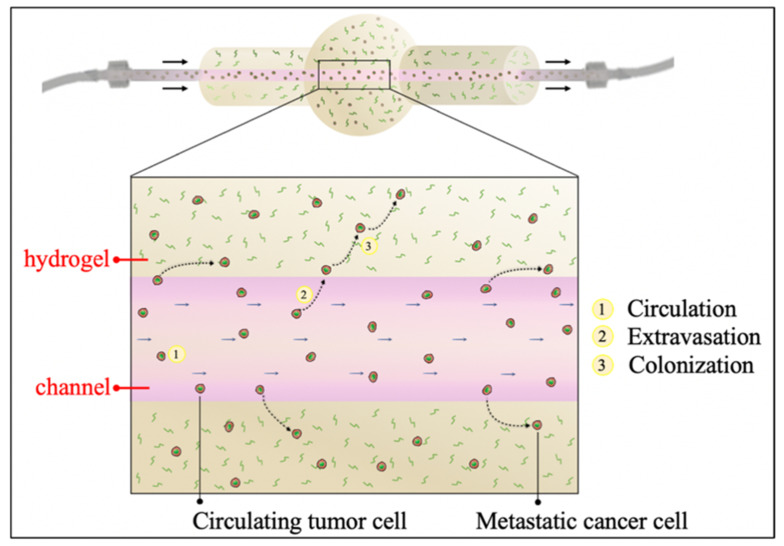
Schematic representation of the main steps of the metastatic cascade.

To obtain a tumor ECM-like model traversed by a perfusable channel, a hydrogel matrix with a single inner vessel-like channel fluidically connected to an external circuit was realized (Figure 1) in order to study the first steps occurring during the metastatic cascade (i.e., cells circulation and survival within the bloodstream, extravasation, spreading and colonization of the metastatic site).

Two kinds of polymers were used and compared to realize the channel-based hydrogel by using two different precursors (alginate solution and fibrinogen, respectively). The polymers were respectively poured into a mold after the insertion of a 21G-needle in an appropriate support. The mold was necessary to create and mechanically sustain the hydrogels. Once alginate or fibrin were cross-linked, the needle was gently removed from the mold leaving cylindrical cavities within the hydrogel. 

Different fabrication parameters (polymeric precursor and cross-linking agent concentrations, precursor/cross-linking agent ratios) were tested for the two polymers, respectively. As result, we observed that highest concentration of fibrinogen (40 mg/mL) and alginate precursor in a weight/volume ratio higher than 1.5% were difficult to pour into the mold due to the high viscosity of the solutions. On the other hand, the hydrogel collapsed by using alginate concentrations lower than 1.5 % *w*/*v*. Accordingly, we chose alginate 1.5% *w*/*v* and fibrinogen 20 mg/mL as precursor concentrations. Regarding the cross-linker agent, 1 M CaCl_2_ was selected, to guarantee performant mechanical properties without impairing cells viability [43]. Thrombin concentration of 10 U/mL was chosen because it was enough to enable a good manipulation of the final hydrogel. Therefore, the final protocol of alginate realization consisted of alginate 1.5% *w*/*v*, CaCl_2_ 1 M and a CaCl_2_/alginate ratio of 1:3 *v*/*v*, whereas the fibrin hydrogel was prepared by mixing fibrinogen 20 mg/mL and thrombin 10 U/mL at 1:1 *v*/*v* ratio. 

### 3.2. Hydrogels Characterization

Firstly, the chemical-physical characteristics of alginate and fibrin hydrogels without channel were assessed. To successively examine the glucose distribution within the hydrogels, experimental glucose diffusion measurements were carried out, by performing a CFD mass transport analysis. The mass transport process occurring between the medium and the hydrogel was simulated by using Comsol Multiphysics in order to obtain the best fitting of the raw data (not shown) and thus to identify the optimal glucose diffusion coefficient. Results are reported in Table 1.

Subsequently, both polymers were mechanically characterized: the storage and viscous moduli were evaluated by performing a DMA analysis at a physiological frequency (1 Hz). 

Results reported in Table 1 show the elastic and viscous moduli for both the hydrogels. The alginate elastic and viscous moduli resulted in being considerably higher than the fibrin ones.

The elastic modulus values are directly correlated with the degree of crosslinking of the polymeric compounds. It can be observed that the crosslinking density of the alginate was higher than the fibrin one, reflecting the different mechanical properties of the two polymers.

The damping properties, represented by the Loss factor, showed that for both the hydrogels the elastic component was more dominant than the viscous one (loss factor << 1). This means that these materials can be considered simple linear elastic ones. Accordingly, the Young modulus was used as input hydrogel parameter in the following computational simulations. 

**Figure 2:**
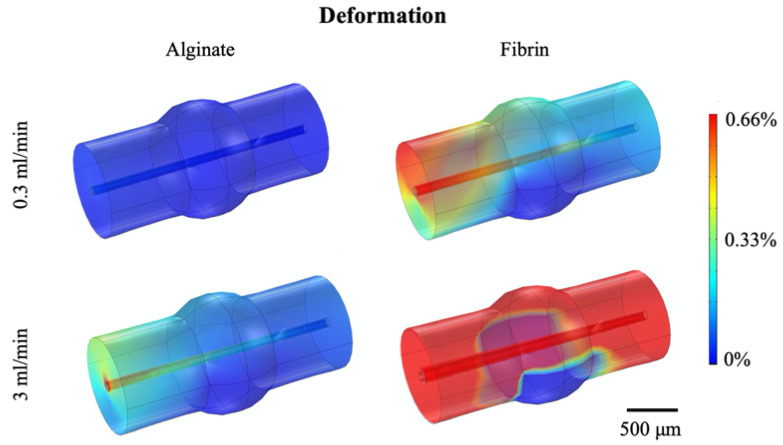
Channel displacement within alginate and fibrin models evaluated at minimum flow rate (0.3 mL/min) and maximum flow rate (3 mL/min).

These values were used as input in the further CFD simulations, performed to predict the mechanical response of the channel-based hydrogels in presence of a fluid flow. In particular, Newtonian incompressible fluid was considered and, according to the theoretical analysis Equation (7), the 0.3–3 mL/min range was imposed as input flow rate for the CFD simulations. In particular, the fluid-dynamic load transmitted from the fluid to the hydrogel was quantified through the FSI Multiphysics. Alginate or fibrin channel-based hydrogels were modeled as linear isotropic elastic materials, as results of the mechanical characterization (Table 1). The radial channel displacement was compared to the initial diameter size (800 μm) to evaluate the channel deformation respect to its initial dimensions. Both polymers showed a low channel deformation and to properly withstand the fluid flow-dynamic load. In particular, it can be noticed in Figure 2 that the fluid flow-associated stress and the resulting deformation were higher at the beginning of the channel, both for alginate and fibrin, showing that such area is crucial to ensure a fluidic continuity between the hydrogel and the external circuit.

**Figure 3:**
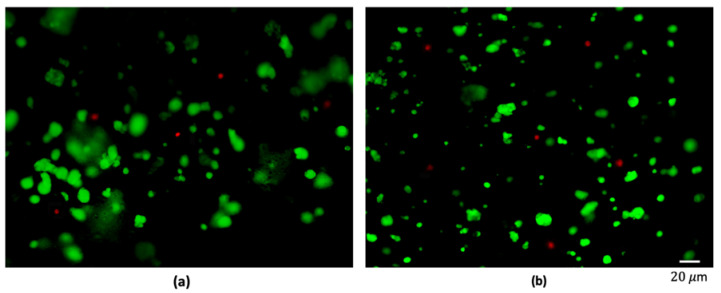
Live/dead staining on MDA-MB-231 cultured in channel-based alginate (**a**) and fibrin (**b**) hydrogels.

Finally, alginate and fibrin 3D samples were tested and compared to evaluate their biocompatibility. Breast cancer cells from adenocarcinoma (MDA-MB-231) were embedded within the two polymers. After 24 h of culture, cells viability was evaluated by using the live/dead assay to discriminate live and dead cells, revealing a cell viability higher than 90% in both cases (93.5% ± 2.2% for alginate hydrogels and 95.7% ± 1.2% for fibrin ones). Moreover, cancer cells were characterized by a round shape typical of the in vivo native tumor environment, as shown in Figure 3. Despite the different chemical composition and mechanical properties of the two polymers, no substantial differences were highlighted in cells viability and organization within the matrix, confirming that alginate and fibrin are both suitable candidates to mimic the tumor extracellular surrounding environment, as widely documented in the literature [44,45] and that our protocol of hydrogels fabrication is not harmful for cells’ viability. 

### 3.3. Perfusion Tests

**Figure 4:**
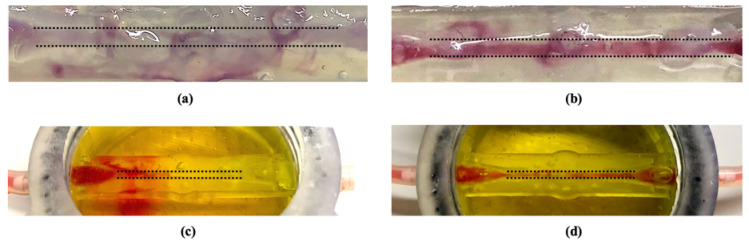
Hydrogels perfusion tests. The dotted line indicates where the channel is located. Comparison between alginate (**a**) and fibrin (**b**) capability to withstand the fluid passage at the same flow rate (1 mL/min); comparison between 3 mL/min (**c**) and 1.5 mL/min (**d**) as input flow rate values for fibrin hydrogel.

Perfusion tests were performed to experimentally compare the capability of alginate and fibrin to withstand the fluid flow at the same flow rate. According to the theoretical and CFD analyses, the 0.3–3 mL/min range, corresponding to a WSS of 1 Dyn/cm^2^ and 10 Dyn/cm^2^ respectively, was imposed as input flow rate at the pump level. Fibrin revealed to be the most performing material. In fact, as it is shown in Figure 4b, the dye flowing into the channel, at flow rate values equal or lower than 1 mL/min, did not disperse within the fibrin polymeric matrix, differently from in presence of alginate (Figure 4a), where the fluid rapidly permeated from the channel to the hydrogel, Moreover, it was possible to carry out tests up to 24 h by using fibrin, whereas alginate collapsed after only a few hours. 

Therefore, further perfusion tests only on fibrin hydrogels were executed to experimentally investigate effects of different input flow rates in the established range. As the FSI analysis predicted, the first tract of the channel was confirmed to be the critical point for the fluidic continuity with the external circuit, since liquid leakages occurred only in this area. In addition, as it is shown in Figure 4c, flow rate values higher than 1.5 mL/min, therefore higher than 5 Dyn/cm^2^ of WSS, often caused channel damaging, yielding to a liquid leakage. 

On the other hand, a perfect fluidic continuity was obtained by setting a flow rate of about 1.5 mL/min (Figure 4d), corresponding to a WSS of 5 Dyn/cm^2^, as CFD simulations predicted (Figure S1 from the Supplementary Materials), which is a physiological micro-vessel WSS [25].

### 3.4. Glucose Diffusion Analysis

**Figure 5:**
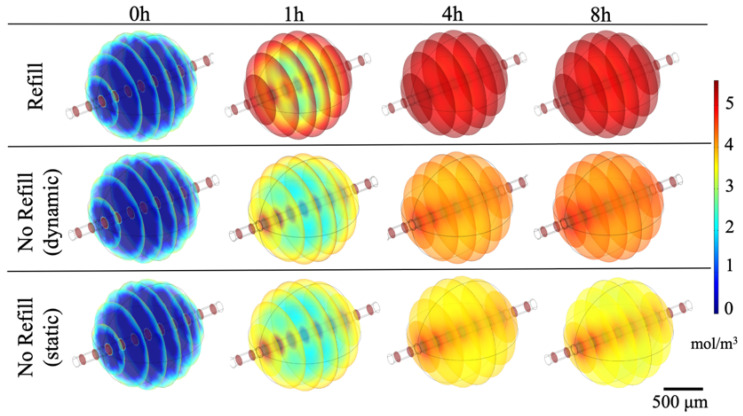
Glucose concentration within fibrin hydrogels modeled simulating three different scenarios: (i) continuous culture medium refilling, (ii) dynamic no culture medium refilling, (iii) static no culture medium refilling within the chamber. Four different time points are reported: t_0_ = 0 h, t_1_ = 1 h, t_2_ = 4 h, t_3_ = 8 h.

Two different culture conditions were simulated by employing the TDS module of COMSOL Multiphysics 5.5: the so-called “continuous refill” culture condition, characterized by a continuous medium refresh over time, and the “no refill” condition. In addition, the static and dynamic scenarios for the “no refill” condition were investigated. The simulation was carried out up to 8 h, by using as input values the physical parameters found by performing the experimental absorption studies (Table 1). Figure 5 shows glucose distribution over time for all the culture conditions. A homogeneous spatial distribution in the refill condition just after 4 h was achieved, whereas in the other scenarios (no refill, both static and dynamic) a higher glucose accumulation around the vessel can be observed, since in these cases the nutrients renewal could occur only through the fluid flow within the channel. 

**Figure 6:**
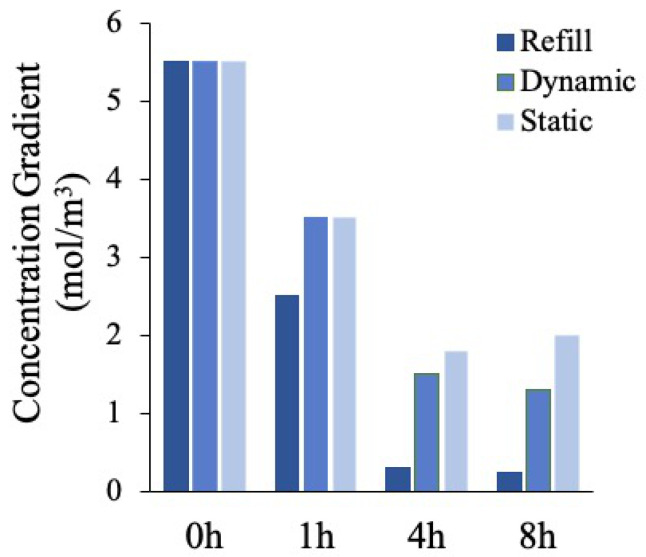
Glucose concentration gradients values in the three culture scenarios considered at different time points: t_0_ = 0 h, t_1_ = 1 h, t_2_ = 4 h, t_3_ = 8 h.

Finally, we calculated the glucose concentration gradient as the difference between the maximum and the minimum glucose concentration values for each time point considered (Figure 6). If the glucose is not homogeneously distributed, the concentration gradient is higher, since this last is representative of the concentration discrepancies within the polymeric matrix. The results reported confirm the trends previously described. 

In summary, a constant monotone decreasing glucose gradient was observed in the refill and dynamic no refill mode, meaning that the glucose was uniformly delivered to the cells in the gel over time, whereas in the static condition, an initial decrease, due to the initial large disparity between the surrounding culture medium rich in glucose and the glucose-free polymer matrix, was followed by a gradient increase, indicating a non-homogenous glucose profile. 

### 3.5. Recovered Cells Number and Viability after Circulation 

**Figure 7:**
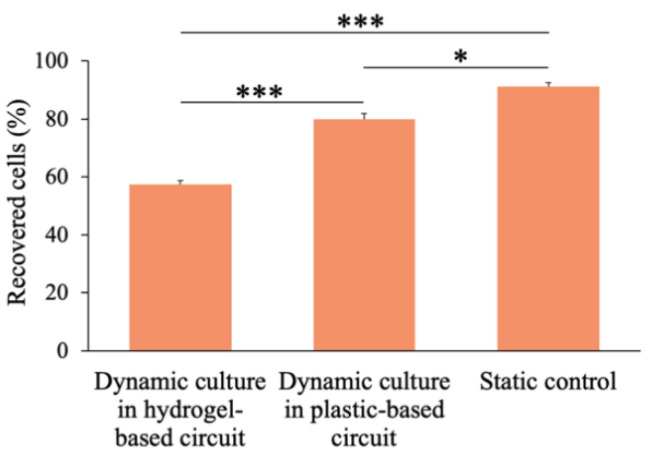
Number of recovered cells from the hydrogel-based circuit, the plastic tube-based one and the static control after 24 h. Values are reported as mean ± s.d., *N* = 3, student’s t-test: * *p* < 0.05, *** *p* < 0.001.

High metastatic potential breast adenocarcinoma cells (MDA-MB-231) circulated within the hydrogel-based fluidic platform under physiological WSS (5 Dyn/cm^2^) conditions. Cell suspension was collected after 24 h of circulation from the circuits and cells were counted. The percentages of recovered cells from the hydrogel-based circuit, the plastic tube one and the static control, respect to the total number of cells injected, were 57.5% ± 1.3%, 80.1% ± 1.9%, 91.3% ± 1.2%, respectively (Figure 7).

**Figure 8:**
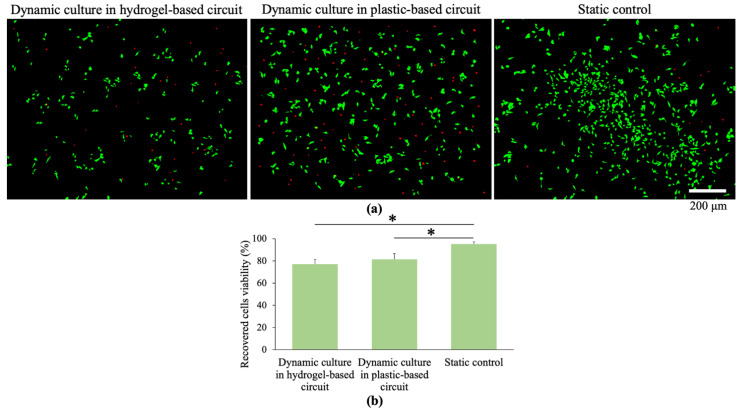
Recovered cells viability measured by live/dead staining of cells after 24 h of circulation within the hydrogel-based circuit (WSS = 5 Dyn/cm^2^), plastic-based circuit (dynamic control, WSS=5 Dyn/cm^2^) and culture within petri dish (static control) (**a**); quantitative analysis of the images, values are reported as mean ± s.d., *N* = 3, student’s t-test: * *p* < 0.05 (**b**).

After counting the recovered cells, they were plated in 96 well plates and cultured to allow their adhesion. After 24 h, the supernatant containing the dead cells, which were not able to adhere on the plastic surface (or detached), was collected, while a fluorescent staining (live/dead) was performed on the remaining adherent cells (Figure 8).

Image post-processing analysis of fluorescent images (Figure 8a) revealed a cell viability of 77.3% ± 4.1% after cells circulation within the hydrogel-based circuit under a WSS of 5 Dyn/cm^2^, and of 81.3% ± 5.3% at the same WSS 5 Dyn/cm^2^ in the hydrogel-free plastic circuit, whereas in the static control resulted equal to 95.2% ± 1.9%. This means that the WSS affected cells viability, although at this physiological value (5 Dyn/cm^2^) the cells viability was still high (around 80%) after 24 h of culture.

It is important to note that the number of dead cells which did not adhere on the well plates after recovery from the circuits was not significantly different (data not shown) in all cases suggesting that the cells viability is not altered respect that represented in Figure 8. 

Interestingly, no significant differences in cells viability were found after cells circulation in the fluidic platform with and without the hydrogel.

The lower number of cells recovered in the hydrogel-based circuit could be due to the presence of a bioactive fibrin surface where breast cancer cells could adhere. Therefore, we then investigated the presence of cells within the hydrogel, to evaluate if they migrated toward the fibrin.

### 3.6. Cells Migration within the Hydrogel under Fluid Flow

**Figure 9:**
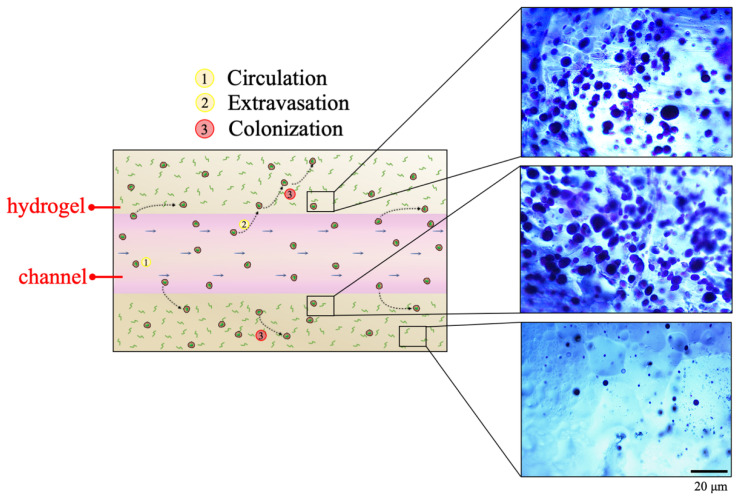
Toluidine blue staining on fibrin hydrogel identifying cells extravasated and migrated within the fibrin hydrogel.

CTCs migration within the hydrogel-based surrounding environment was qualitatively evaluated by performing a toluidine blue staining on the fibrin hydrogel. As can be observed in Figure 9, MDA-MB-231 extravasated from the channel and migrated in the hydrogel. The high presence of embedded cells within the fibrin hydrogel confirmed that the difference between the absolute number of cells recovered from the hydrogel-based circuit and from the hydrogel-free one was due to the cells migration toward the hydrogel. In particular, most of the migrated cells were found in the polymeric matrix portions closer to the channel where they circulated.

## 4. Discussion

Cancer metastasis represents one of the leading causes of death. Despite recent advances in cancer research, the establishment of a reliable in vitro platform to accurately explore the crucial mechanisms involved during the metastatic dissemination remain still a challenge. To meet this need, here we realized a novel in vitro fluidic platform to deeply investigate the cancer cells extravasation and colonization in a 3D hydrogel-based metastatic model under physiological hematogenous forces. 

Firstly, a 3D printer was employed to fabricate a plastic mold to realize 3D hydrogels provided with an inner channel directly connected to an external fluidic circuit. Alginate and fibrin were tested and compared as bulk polymers for the hydrogel realization. In particular, hydrogels fabrication protocols were optimized to obtain a balance between the feasibility of the channel-based system and the performance of the final hydrogel-based structure. Specifically, the two polymeric concentrations were optimized to be low enough to be easily handled in the molds and high enough to guarantee a suitable stiffness of the final hydrogels, able to withstand the hydraulic pressure generated by the fluid flow. In fact, mechanical properties are fundamental at the macroscale level as well as at the microscale one. In this context, it is widely recognized that the mechanical properties condition cell activity [46]. For example, breast cancer cells showed a decreased viability by increasing alginate hydrogels elasticity, while soft hydrogels (E < 200 kPa) were more permissive to cell proliferation [44]. Here, DMA results reported that alginate and fibrin hydrogels stiffness was in this range. Besides the biocompatibility, it was important to predict, through CFD simulations, the hydrogels mechanical response to external fluidic stimuli. In fact, fluid flow-derived stimulation within compliant and deformable materials, such as soft hydrogels, may lead to structural deformations [47]. FSI results indicated that both polymers endured the flow-associated stresses, preventing matrix collapses and liquid leakages of the circulating medium, by imposing a flow rate range of 0.3–3 mL/min, corresponding to the physiologic WSS of 1–10 Dyn/cm^2^ within the channel. In fact, such WSS values establish a physiologically relevant range of fluid forces experienced by CTCs in microvascular vessels [48]. Furthermore, it is commonly accepted that fluid flow patterns have a regulatory effect on tumor cells adhesion, proliferation, and invasion. In particular, the shear stress can influence the CTCs survival during the vascular transport and thus their metastatic potential [25]. Therefore, it is important to resemble the cancer hemodynamics, especially the biophysical properties experienced by the CTCs during the metastatic migration in the bloodstream.

As first platform validation, experimental perfusion tests were carried out by fluidically connecting the hydrogels to the external fluidic circuit to verify the real capability of the 3D hydrogels to withstand the fluid flow stimuli. Interestingly, results revealed that fibrin was the most performing material, since liquid dispersion within the matrix was absent and the channel was clearly visible after perfusion. This behavior can be due to the well-known adhesive properties of the fibrin that make it widely adopted in the surgical field as a sealant to promote tissues bonding [49,50].

Another major current challenge in designing 3D hydrogel-based culture systems relies on the mass transport limitations experienced when using clinically relevant length scales (centimeters) [51]. In fact, one of the major common limitations of the hydrogel-based constructs is represented by the passive cell culture medium diffusion, which is inadequate to deliver nutrients over large length scale, thus resulting in nutrient and waste discrepancies within the constructs, reducing cell viability as well as altering cellular phenotypes [52,53]. 

In fact, ideally, once the CTCs have colonized the hydrogel representing the metastatic site, they need an adequate nutrients supply, present in the culture medium, to carry on their metabolic activity. Therefore, we simulated three different scenarios: the medium “continuous refill” culture condition, characterized by a continuous medium refresh over time in the culture chamber and the “no refill” condition, in static and in dynamic culture, respectively. As reported, in each of these configurations, an initial (first 4 h) increase of the glucose concentration inside the hydrogel occurred, due to a massive medium diffusion to the gel, caused by the high glucose concentration difference between inside (0 mol/m^3^) and outside (5.5 mol/m^3^) the polymer. After 4 h, a change in the trend occurred for the “no refill” static scenario. In fact, a small decrease of glucose concentration was shown in the following h (up to 8 h), resulting in higher concentration inhomogeneities within the entire matrix. On the contrary, for the “no refill” dynamic culture mode, an enhanced glucose spatial uniformity within the hydrogel was reached, demonstrating that the dynamic stimuli had a clear effect on improving the hydrogel glucose supply due to the additional convection process occurring within the system. Accordingly, the “no refill” dynamic scenario was more similar to the “continuous refill” one, which represented the ideal culture condition.

Finally, once the platform was fluidically validated and the nutrient transport mechanisms optimized, highly metastatic breast cancer cells (MDA-MB-231) were injected within the circuit and exposed to physiological WSS conditions of 5 Dyn/cm^2^, to assess their viability upon circulation as well as their capability to migrate toward the hydrogel and colonize the metastatic model. Since a lower number of cells was recovered from the hydrogel-based circuit (57.5% ± 1.3%), respect the hydrogel-free one (80.1% ± 1.9%), we supposed that this remarkable difference was due to the presence of the bioactive fibrin [49], where breast cancer cells could adhere. Hence, we investigated the presence of cells within the hydrogel, to evaluate if they migrated toward the fibrin. As Figure 9 shows, a large presence of cells within the polymeric matrix can be shown, confirming that invasion and adhesion to the hydrogel occurred under physiological WSS conditions. 

Moreover, it is well-known that vasculature structure plays a pivotal role on CTCS extravasation and that the complex interplay between the ECs and cancer cells needs to be reproduced to fully elucidate the mechanisms of cells invasion in a secondary tissue [28,54]. Furthermore, the endothelium is susceptible to different shear stresses by altering morphology, gene expression profiles, ECs-ECM interactions and barrier transport capabilities [25]. Based on these considerations, further studies could be performed aimed at increasing the level of accuracy of our fluidic platform by associating multiple components typical of the metastatic niche (e.g., chemical signals, cells recreating the tumor stroma, endothelial cells to re recreate the endothelial barrier functions). In addition, biomolecules, as vascular endothelial growth factor (VEGF), can be incorporated in our system, since, for example, VEGF gradients together with shear forces may promote ECs sprouting and vasculogenesis of capillary-sized networks within the ECM [28,55]. 

Here, we realized a 3D hydrogel-based fluidic platform by coupling an experimental approach to an in silico analysis to assess the optimal culture conditions and precisely control fluid patterns within a ECM-based channel, thus providing the scientific community a low-cost and efficient in vitro model to carry out systematic studies on the development and progress of the metastatic process.

## 5. Conclusions

Cancer metastatic dissemination to a secondary site represents one of the leading causes of death worldwide. Blood flow-associated stimuli play a pivotal role on CTCs vascular transport, affecting cancer diffusion to distant tissues. Therefore, a new hydrogel channel-based system was realized and fluidically connected to an external fluidic circuit, as a preliminary 3D in vitro model, to mimic a secondary metastatic site where CTCs can migrate under a proper hemodynamic cancer environment. Once fluid-dynamic conditions and nutrients transport kinetics were optimized, the effects of physiological WSS and fluid flow on breast cancer cell line were assessed. A slight decrease in cells viability due to the fluid flow and the cells migration and colonization of the hydrogel were observed.

These outcomes indicate that our perfusable hydrogel-based system is a valid model to in vitro study the behavior of detached CTCs in circulation and to investigate some mechanisms related to the metastatic cascade that still remain unknown. In particular, further works will be aimed at improving the realism and complexity of the fluidic platform, by resembling, for example, the vascular barrier affecting the CTC adhesion to the metastatic site, or the coculture of mesenchymal/endothelial cells and tumor cells in the hydrogel models toward a higher reliability of the tumor niche. The system could be then adopted for testing in vitro the efficacy of anti-metastatic drugs and therapeutic strategies in pre-clinical assays, through the 3R paradigm. In particular, this approach may allow performing drug efficacy studies in a fluid-dynamic environment, which is the natural domain for CTCs. The capability of the metastatic cells to survive and colonize a tissue could be monitored, evaluating the degree of CTCs penetration at a secondary site. Moreover, the high versatility of the platform will allow reproducing many different types of cancers that can give origin to metastatic events.

## Figures and Tables

**Table 1:** Hydrogels chemical-physical characteristics. Data are reported as mean ± s.d.

Material	Elastic Modulus [kPa]	Viscous Modulus [kPa]	Loss Factor	Crosslinking Density [mol/cm^3^]	Diffusivity [m^2^/s]
Alginate	167.3 ± 9.0	30.4 ± 0.5	0.18 ± 0.01	2.2 ± 0.1 · 10^−5^	5.0 ± 0.8 · 10^−10^
Fibrin	7.2 ± 0.8	0.9 ± 0.1	0.13 ± 0.02	9.7 ± 1.1 · 10^−7^	4.4 ± 0.5 · 10^−10^

## Supplementary Materials

The following are available online at https://www.mdpi.com/2073-4360/12/11/2467/s1, Figure S1: Simulated SS profiles across the channel at minimum (0.3 mL/min), medium (1.5 mL/min) and maximum flow rate (3 mL/min) imposed according to the theoretical analysis.
