# Supplementary material for: 3D Perfusable Hydrogel Recapitulating the Cancer Dynamic Environment to in Vitro Investigate Metastatic Colonization
Source: Polymers (Basel). 2020 Oct 24;12(11):2467. doi: 10.3390/polym12112467 (PMC7690854; doi:10.3390/polym12112467)
Supplement: Supplementary file 1 [file polymers-12-02467-s001.pdf]

Supplementary Materials

3D Perfusable Hydrogel Recapitulating the Cancer Dynamic Environment to in Vitro Investigate Metastatic Colonization

Supplementary Figures and Tables:

Computational simulations to finely assess the fluid flow properties within the channel were performed. The analysis was conducted by employing the Single-Phase Laminar Fluid Flow model of Comsol Multiphysics 5.5 assuming i) a laminar flow regime, ii) an incompressible Newtonian fluid. Specifically, in order to obtain WSS values comprised in the physiological range (1–10 Dyn/cm^2) according to the theoretical analysis (Equation 5), the SS distribution (Figure S1) within the channel was estimated since it is the main fluid flow-associated force influencing CTCs and region-specificity for tumor metastasis [1].

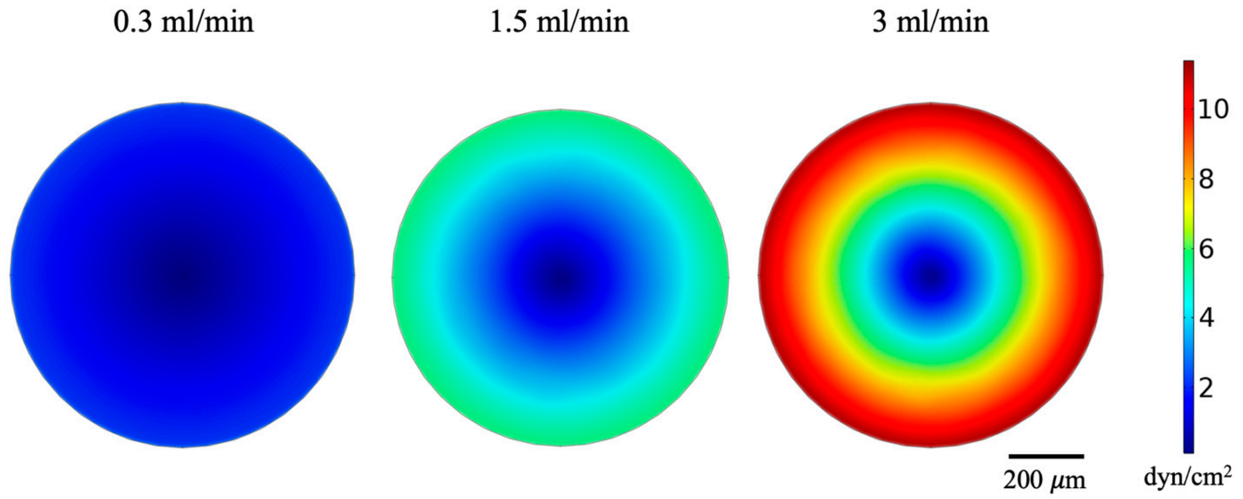

Figure S1. Simulated SS profiles across the channel at minimum (0.3 mL/min), medium (1.5 mL/min) and maximum flow rate (3 mL/min) imposed according to the theoretical analysis.

References

1. Buchanan, C.F.; Verbridge, S.S.; Vlachos, P.P.; Rylander, M.N. Flow shear stress regulates endothelial barrier function and expression of angiogenic factors in a 3D microfluidic tumor vascular model. *Cell Adhes. Migr.* **2014**, *8*, 517–524, doi:10.4161/19336918.2014.970001.
